# Protocol to rapidly screen CRISPR-Cas9 gene editing outcomes in a cell population by mutating eGFP to a blue or non-fluorescent phenotype

**DOI:** 10.1016/j.xpro.2025.103950

**Published:** 2025-07-15

**Authors:** Danny Wilbie, Enrico Mastrobattista, Olivier Gerrit de Jong

**Affiliations:** 1 Department of Pharmaceutics, Utrecht Institute for Pharmaceutical Sciences (UIPS), Faculty of Science, Utrecht University, Utrecht, the Netherlands

**Keywords:** Cell-based Assays, Gene Expression, CRISPR

## Abstract

When designing genome editing therapy, it is crucial to measure outcomes of DNA damage repair. Here, we present a protocol to distinguish the outcome of targeted DNA damage repair from the bottom up, through a previously established readout of enhanced green fluorescent protein (eGFP) to blue fluorescent protein (BFP) mutations. We describe steps for producing eGFP-positive cells and differentiating between non-homologous end joining-induced gene knockout and homology-directed repair-induced-directed mutation in these cells. This protocol has potential for high-throughput and scalable assessment of gene editing techniques.

For complete details on the use and execution of this protocol, please refer to Walther et al.^1^ and Wilbie et al.^2^

## Highlights

• Instructions for generation of eGFP-positive cell lines via lentiviral transduction
• Steps for transfection of gene editing reagents in eGFP-positive cells
• Steps for cell handling post-transfection and measuring cell fluorescence using FACS
• Guidance on FACS data processing, analysis, and interpretation of gene editing outcomes

## Abstract (editor highlights)

Publisher’s note: Undertaking any experimental protocol requires adherence to local institutional guidelines for laboratory safety and ethics.

## Graphical abstract

**Figure:**
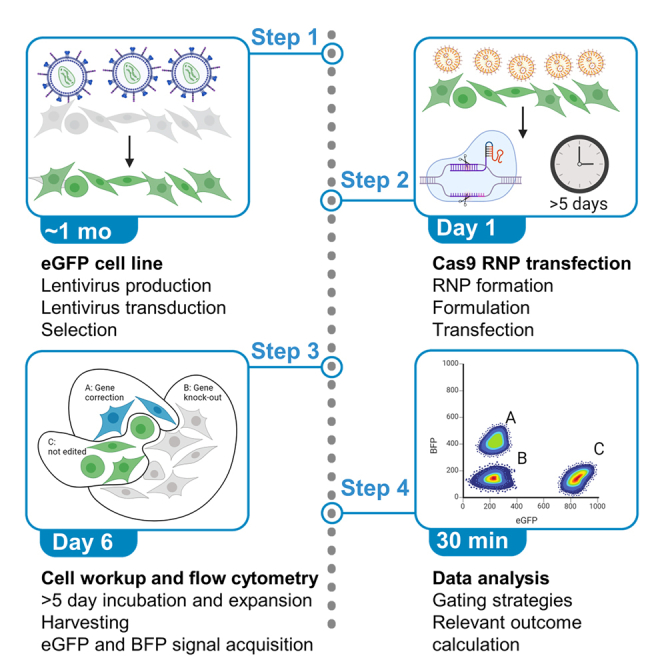

## Before you begin

Genome editing using CRISPR-Cas9 can induce specific gene correction or disruption through activation of different DNA damage repair pathways.^3^^,^^4^^,^^5^ The Cas9 enzyme forms a ribonucleoprotein (RNP) complex with a guide RNA molecule, the sequence of which can target specific genes using ∼20 nucleotides of homology to the genomic target. The RNP induces a blunt double stranded break (DSB) upon recognition and binding of the guide RNA to its target DNA sequence, and the protein-specific protospacer adjacent motif (PAM) in the target DNA to the PAM-interacting domain in the Cas9 protein.^6^ Staggered DNA breaks with overhangs can be generated as well by Cas12a enzymes^7^ and other gene editing mechanisms can be achieved by Cas9-fusion proteins such as base editing and prime editing.^8^^,^^9^

Cells have evolved complex mechanisms to repair DSBs, including those induced by CRISPR-Cas. DSBs are primarily resolved through the non-homologous end-joining (NHEJ) pathway, which anneals the broken DNA strands with a small chance of DNA insertions and deletions at the DSB site. Perfectly repaired DNA can be targeted by CRISPR-Cas again, which leads to a cumulatively high chance of mutations at the target site.^10^^,^^11^^,^^12^ Alternatively, DSB can be repaired by homology-directed repair (HDR), which resects the DNA strands and uses a template DNA molecule to guide the repair. In nature, this is done by the sister chromatid during mitosis, however this process can be hijacked by delivering a synthetic DNA template to induce specific mutations. Small therapeutic mutations are often encoded on single stranded oligo deoxynucleotides (ssODN). In the context of CRISPR-Cas-mediated (therapeutic) mutations, the PAM site is often also mutated to ensure that the DNA is no longer cleavable by SpCas9. A high-throughput method to simultaneously study these repair mechanisms is a valuable tool to employ, as it would allow rapid investigation of many research questions surrounding CRISPR functionality, as well as delivery. Such a method was described by Glaser et al. in 2016, which is expanded and applied in this work with practical guidelines for utilization in a laboratory without prior genome editing experience.^13^

The protocol below describes the generation of eGFP-positive HEK293T cells and an example experiment in which the length of the HDR template was optimized for HDR efficiency. By mutating two amino acids in the eGFP sequence using two single nucleotide polymorphisms, it is possible to shift the fluorescence of the encoded green fluorescent protein to blue fluorescence. Insertions and deletions at this locus lead to loss of fluorescence.^13^ In this way we can differentiate between the activation of either HDR or NHEJ respectively. We have performed this assay using HEK293T, Hepa 1-6, IMR90 and HepG2 cells. This reporter system allows for rapid *in vitro* development of formulations for CRISPR-Cas9 delivery, as well as functional screening of CRISPR-enhancing therapies.^1^^,^^2^ This lowers the barrier to entry and the complexity of CRISPR therapy development, allowing more groups to join the research effort on curing genetic diseases and unraveling the mechanisms that drive genome correction. By using an easily accessible model such as eGFP, these efforts are comparable between studies and may be easier to interpret on the fundamental level before application to specific diseases or models.

### Cell culture preparation

1. Preparation of HEK293T cells.
  a. Thaw a frozen stock of HEK293T cells.
  b. Resuspend the HEK293T cells in 10 mL complete cell culture medium in a 15 mL conical tube (see also materials and equipment setup).
  c. Centrifuge the cells for 5 minutes at 300 x g. Gently remove the supernatant.
  d. Resuspend the cell pellet using 15 mL complete cell culture medium.
  e. Culture the HEK293T cells in a T75 culture flask.
  f. Incubate at 37°C and 5% CO_2_ for at least a week before starting the lentivirus production to ensure efficient cell growth.
  g. If the confluency becomes higher than 80%., passage the cells as described under Routine cell culture steps for HEK293T cells.

***Note:*** Depending on the target cell line of interest, additionally prepare that with a suitable thawing protocol. In this work HEK293T will be used at a low passage number for as a recipient cell line for the transduction as well.

2. Routine cell culture steps for HEK293T cells.
  ***Note:*** These steps are to be repeated every 3–4 days to ensure growth of the cells. The example given here is for a T25 culture flask (25 cm^2^ surface area). Scale up the noted volumes as needed for larger cell culture flask surface areas.
  a. Aspirate cell culture medium from the flask.
  b. Wash cells with 5 mL sterile 1X PBS.
  c. Aspirate the PBS from the cell monolayer.
  d. Add Trypsin-EDTA solution to detach the cells, typically 0.5 mL for a T25 culture flask.
  e. Incubate the cells at 37°C for 2–5 minutes.
  f. Check detachment of cells under the microscope.
  g. Add 2,5 mL of complete culture medium to the cells to inhibit the trypsin and gently resuspend the cells until a single-cell suspension is achieved (for example, mix by pipetting 10x up-and-down using a sterile serological pipette).
  h. Passage a volume to a fresh cell culture flask to allow growth over the next 3–4 days (typically 1:10, two times per week for HEK293T cells).
  i. Add 5 mL of fresh cell culture medium to the new flask.

## Key resources table

**Table:** 

REAGENT or RESOURCE	SOURCE	IDENTIFIER
**Chemicals, peptides, and recombinant proteins**		

SpCas9-NLS	Walther et al.^1^	N/A
Polyethylenimine (PEI), linear, MW 25,000, transfection grade	Polysciences	23966
ProDeliverIN CRISPR	OZ Biosciences	PIC0500
Dulbecco’s modified Eagle’s medium, high glucose	Merck	D5671
Trypsin-EDTA solution	Merck	T4049
Dulbecco’s phosphate-buffered saline (PBS) without Mg2+ and Ca2+	Merck	D8537
Puromycin	InvivoGen	Ant-pr-1
Antibiotic-antimycotic solution 100x	Merck	A5955
Fetal bovine serum	Biowest	S1810-500
Opti-MEM reduced serum medium	Fischer Scientific	11520386
Paraformaldehyde	Merck	158127
Bovine serum albumin	Merck	A9418

**Experimental models: Cell lines**		

Human: HEK293T cells	ATCC	CRL-3216

**Oligonucleotides**		

Optimized BFP mutation template: caagctgcccgtgccctggcccaccctcgt gaccaccctgAGCCACggcgtgcagtgcttcagccgctaccccgaccacatgaagc	Merck	N/A
sgRNA against eGFP locus: GCUGAAGCACUGCACGCCGU	Merck	N/A

**Recombinant DNA**		

pMD2.G	Trono et al.	Addgene #12259
pRSV-Rev	Trono et al.^14^	Addgene #12253
pMDLg/pRRE	Trono et al.^14^	Addgene #12251
pHAGE2-Ef1a-eGFP-IRES-PuroR	De Jong et al.^15^	N/A

**Software and algorithms**		

FlowLogic		https://www.inivai.com/flowlogic
GraphPad Prism		https://www.graphpad.com/

**Other**		

TC20 cell counter	Bio-Rad	1450102
BD FACS Canto II	BD Life Sciences	N/A
Laminar flow hood	N/A	N/A
Nikon ECLIPSE ti2-U inverted and fluorescent microscope	Nikon	N/A
Yokogawa CV7000S spinning disk confocal microscope	Yokogawa	N/A
Sigma 4-16KS refrigerated benchtop centrifuge	Sigma Centrifuges	N/A
Cell culture incubator	N/A	N/A

## Materials and equipment

### Complete cell culture medium

To 500 mL of DMEM, add 55,6 mL of FBS to achieve a v/v concentration of 10% FBS.

### Antibiotic-antimycotic cell culture medium

To 49.5 mL of complete cell culture medium, add 0.5 mL of 100X antibiotic-antimycotic solution to achieve 1X antibiotic-antimycotic solution in cell culture medium.

### Selection cell culture medium

To 50 mL of complete cell culture medium, add 10 μL of Puromycin (10 mg/mL) to achieve 2 μg/mL of puromycin.

### Fixation buffer

Dissolve 1% w/v (for example 1 g/100 mL) of paraformaldehyde powder in Dulbecco’s PBS. Heat to 60 °C. Stir until dissolved (recommended >16 hours). Filter the solution using a 0.45 μm filter.

### 500 mM EDTA

Prepare a stock solution of 500 mM EDTA in ddH2O at pH 8. Sterilize by 0.2 μm filter.

### 1% BSA in PBS

Weigh 1% w/v bovine serum albumin (for example 1 g/100 mL) in Dulbecco’s PBS.

### FACS buffer (1% BSA and 2 mM EDTA in PBS)

Add 1 volume of the 500 mM EDTA stock solution to 250 volumes of the 1% BSA in PBS solution to reach 2 mM EDTA (for example 400 μL to 100 mL). Sterilize the solution by 0.2 μm filter and keep sterile to avoid microbial growth.

## Step-by-step method details

### eGFP lentivirus production

**Timing: 1 week**

This section is aimed at generating eGFP-carrying lentiviral particles and an eGFP-positive cell line as preparation for the actual genome editing assays. These can be acquired commercially as well, skipping these parts of the protocol.

**CRITICAL:** Ensure that the HEK293T producer cells are cultured for at least one week after thawing to allow them to recover. One day prior to starting lentiviral production, passage them to a T25 flask at 30–50% confluency to ensure appropriate confluency during lentivirus production.

1. Prepare pDNA polyplexes.
  ***Note:*** The plasmids noted below are examples of lentiviral production plasmids. Use different lentiviral transfer plasmids that facilitate eGFP expression if desired.
  a. Mix 1.5 μg of psPAX2, 1.5 μg of pMD2.G, and 3 μg of pHAGE-EF1a-eGFP-Puro in 500 μL of OptiMEM without antibiotics in an Eppendorf tube.
  b. Add 18 μg of 25 kDa linear PEI (3 μg PEI per μg DNA used in step 1.a) in a second tube in 500 μL of OptiMEM without antibiotics. Incubate both tubes for 5 minutes at 18°C–24 °C.
  c. Add the contents of the OptiMEM-DNA tube to the OptiMEM- PEI tube and mix gently.
  d. Incubate the tube for 10–15 minutes at 18°C–24°C to allow polyplex formation
2. Add the DNA&PEI mixture directly to the culture medium in the T25 flask containing HEK293T cells.
3. Incubate the cells with the transfection mixture >16 hours at 37°C in a cell incubator.
4. The following morning, aspirate the supernatant from the T25 flask and add 5 mL of warm complete culture medium.

***Note:*** Addition of 1x antibiotic/antimycotic solution at this stage is recommended to avoid bacterial or fungal contamination of the cells.

5. Incubate for an additional 48 hours at 37°C in the cell incubator to allow virus to be produced.
6. Harvest the supernatant containing the lentiviral particles in a 15 mL conical tube.
7. Centrifuge the tube for 5 minutes at 500 x *g* at 18–24°C to remove cells.
8. Collect the supernatant and filter using a 0.45 μm syringe filter into a clean 15 mL tube.

**CRITICAL:** The supernatant can be used directly for transduction, or stored at −80°C until further use. Avoid repeated freeze-thaw cycles as this affects virus integrity and infectivity.

### Lentiviral transduction to generate an eGFP+ cell line

**Timing: 2 weeks**

The aim of this step is to achieve an eGFP+ cell line at a low multiplicity of infection (MOI) to avoid multiple integrations of the eGFP gene, which would complicate gene-editing outcome analysis later on. Therefore the steps below can be done on several conditions using a dilution range of lentiviral supernatant. A low MOI will likely result in 1 – 10% of cells exhibiting eGFP expression prior to antibiotic selection, therefore a low lentiviral efficiency is not necessarily an issue in these experiments.

9. Passage cells to be 50% confluent on the day of transduction in a T25 flask.
10. At 50% confluency, aspirate the cell culture supernatant.
11. Add lentivirus-containing supernatant to the cells. Generally, to reach 1 – 10% eGFP expression, a volume between 50 – 500 μL is required. Fill to 5 mL with complete culture medium if a lower volume of supernatant is used (See also troubleshooting).
12. Incubate the cells >16 hours at 37°C in a cell culture incubator.
13. The following morning, safely remove and discard the cell culture medium, and add 5 mL of fresh complete culture medium.
14. Incubate the cells for 24 hours at 37°C in a cell culture incubator.
15. Evaluate the eGFP expression using fluorescent microscopy.
16. If applicable, add selection antibiotics (e.g. puromycin, in this case for HEK293T cells at a final concentration of 2 μg/mL) to the cell culture medium.

***Note:*** Selection antibiotic concentration will differ for each cell line, and may need to be optimized using a kill-curve experiment prior to selection.

17. Incubate the cells at 37°C in a cell culture incubator.

**CRITICAL:** The puromycin selection will cull non-transduced cells, so the confluency will likely be lower after selection. If many dead cells are observed (floating in the medium), refresh the medium. eGFP expression will be visible after 24–48 h through fluorescent microscopy. If the expression of eGFP is very heterogeneous refer to troubleshooting 1.

18. Expand the cells to appropriate (e.g. T175) numbers for cryopreservation.

### Transfecting CRISPR-Cas9 reagents in cells

**Timing: 1 day (step 2) followed by 30–60 min (steps 3–4)**

In this section the CRISPR-Cas reagents are formulated into lipoplexes using the commercial transfection reagent ProDeliverIN CRISPR, which was suitably cytocompatible and effective in our experience. Example concentrations and volumes are given below to achieve 15 nM RNP and 30 nM HDR template in the well, which is sufficient for robust HDR activation in HEK293T cells as optimized in our previous work.^1^ Other cell lines may require scaling up the concentration and optimizing the relative amounts of protein and template DNA.

19. Calculate the volumes of SpCas9, sgRNA, HDR template and ProDeliverIN CRISPR stocks needed depending on the necessary concentration. Examples are given below for 15 nM RNP in the well.
20. Cell plating.
  a. Culture cells according to normal cell culture protocols.
  b. Harvest cells in an appropriate manner for the used line, e.g. using trypsin/EDTA solution.
  c. Mix 7.5 μL of cell suspension with 7.5 μL of a 0.1% Trypan Blue solution. Add mixture of cells and Trypan Blue to a counting slide and count the cells using an appropriate cell counting method (in our case, we used a TC20 cell counter).
  d. Dilute cells to a suitable confluency for a 96 well plate using completed medium in 100 μL/well. For example: 100,000 cells/mL, resulting in 10,000 cells/well when 100 μL is used. Seed the cells in the 96 well plate.
    **CRITICAL:** Exclude antibiotics from the cell culture medium at this stage. Transfections in the presence of antibiotics may show increased toxicity for certain transfection reagents including ProDeliverIN CRISPR in our experience.
  e. Incubate >16 hours at 37°C in a cell culture incubator.
21. RNP formulation.
  a. Transfer SpCas9 to a 1.5 mL Eppendorf tube, dilute to 2.5 μM using OptiMEM.
  b. Add sgRNA in a 1:1 molar ratio of SpCas9:sgRNA, mix by pipetting.
  c. Incubate the tube at 18°C–24°C for 15 minutes to allow RNP formation.
  d. Dilute the RNP complexes to 0.1 μM using OptiMEM. Mix well by vortexing.
  e. Add the HDR template at a 2:1 ratio of DNA:SpCas9. Mix well by vortexing.
  f. Add ProDeliverIN CRISPR reagent at a ratio of 1 μL ProDeliverIN CRISPR to 2 pmol of SpCas9 protein.
  g. Incubate for 5 minutes at 18°C–24°C.
22. Transfection.
  a. Add 17.7 μL of the transfection mix to each well to reach 15 nM of RNP.^1^
  b. Incubate the cells to allow uptake and transfection of Cas9 RNPs.

**CRITICAL:** Some transfection reagents show toxicity over time. In this case it is recommended to gently wash the cells by replacing the culture medium after 24–48 h. We recommend to default to guidelines of the manufacturer of the transfection reagent.

### Cell workup and flow cytometry

**Timing: 5 days (cell expansion) + 4–8 h (flow cytometry workup and run)**

**Figure 1:**
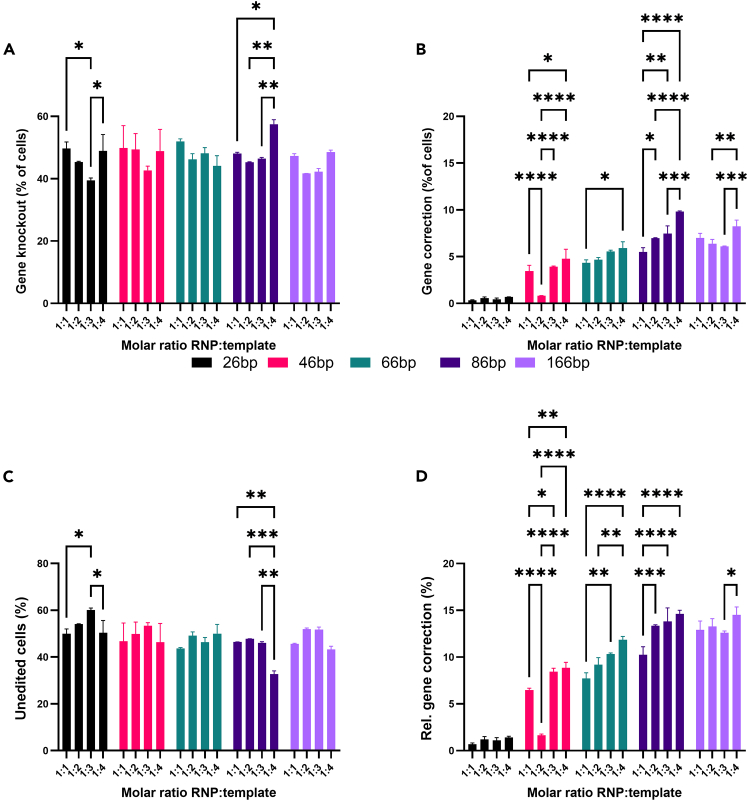
Optimization of HDR template length in HEK293T-eGFP cells Here, using ProDeliverIN CRISPR-Cas9 transfections, the homology arms in the ssODN template were varied in length from 10 nt to 80 nt at both sides of the mutation. The molar ratio of the template to the Cas9 RNP in the formulation was varied as well to optimize relative HDR gene correction. n = 3 technical replicates. (A) Gene knockout caused by these formulations, measured as the percentage of non-fluorescent (eGFP- BFP-) cells. (B) Gene correction caused by these formulations, measured as percentage of blue cells (eGFP- BFP+). (C) Unedited cells, measured as percentage of green cells (eGFP+ BFP-). (D) Relative (Rel.) gene correction incidence, measured as percentage of blue fluorescent cells in all gene edited (GFP-) cells. 2-Way ANOVA with Tukey’s multiple comparisons post-hoc analysis performed is shown. Only significant differences are shown on the graph. ∗ = *p* <0.05, ∗∗: *p* = 0.01, ∗∗∗, *p* < 0.001, ∗∗∗∗, *p* < 0.0001.

To visualize both NHEJ and HDR DNA repair outcomes, an incubation time of up to 5 days is recommended, as shown to be optimized in Figure 1. This is due to the long half-life of eGFP, as well as the production of BFP which needs time to accumulate in the cell. Expansion of cells for several days after the genomic modification does not affect experimental outcome, as the genomic modification is retained when cells divide. Depending on the proliferation speed of the used cell type, it might be required to passage cells to allow further expansion of cells and to avoid over-confluency and cell death.

**Figure 2:**
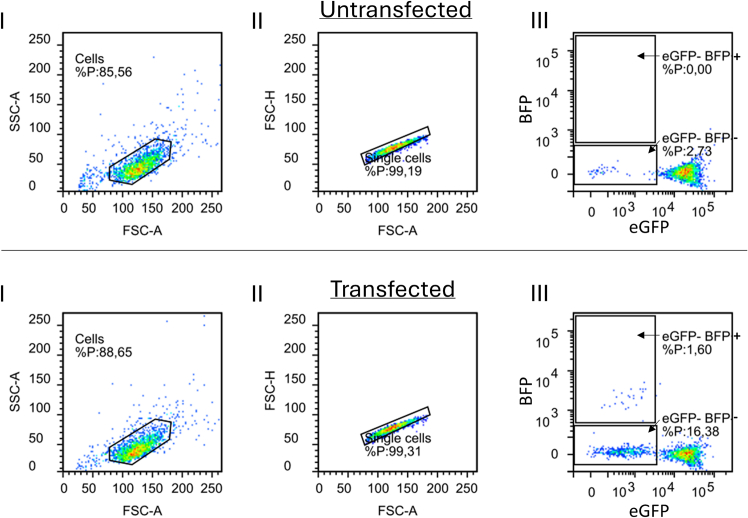
Typical flow cytometry plots for gene editing analysis in HEK293T-eGFP cells Gating for untransfected (top) or Cas9/sgRNA/HDR template transfected (bottom) eGFP HEK293T. Gating is shown for cells (I), single cells (II) and the analysis of the fluorescent populations (III).
    vi. Including measurement of non-fluorescent (untransduced) control cells to accurately gate eGFP and BFP positive cells is recommended.
    vii. Measure samples according to the instrument specifications. Measure at least 10,000 events to achieve a robust population of all conditions.
    viii. Collect the raw .fsc data for analysis.
    ix. Shut down the flow cytometer according to the instrument’s specifications.

23. Cell expansion and maintenance.
  a. Assess the confluency of the cells under the microscope to determine whether cell passaging is required. If so (>80% confluency), proceed to step 2.
  b. Passage the cells from the 96 well plate to a 48 well plate.
    ***Note:*** Other plate formats are suitable as well, a 48 well plate is ∼3x more surface per well than a 96 well plate and as such sufficient for 5 days of culture in our experience.
    i. Aspirate the medium from each well.
    ii. Wash the cells with 50 μL of PBS per well. Avoid directly pipetting onto the cell layer to prevent detachment.
    iii. Add 30 μL of Trypsin-EDTA to all wells.
    iv. Incubate at 37°C to allow cell detachment (5 minutes for HEK293T cells).
    v. Dilute the trypsin in all wells with 80 μL of complete culture medium.
    vi. Resuspend the cell to a single-cell suspension in all wells by pipetting up and down 5–10x.
    vii. Transfer the full volume of each well to a new well in a 48-well plate to allow expansion.
      ***Note:*** This is a 3x dilution based on the well surface areas. For cells doubling once per day, if the cells are <50% confluent this will lead to roughly 1/6 confluency in the new plate which will reach around 100% confluency in 3 days. Change the transferred cell number according to the used cell line and experience.
    viii. Add 400 μL of complete medium.
    ix. Incubate the cells until a total of 5 days post transfection at 37°C. Cells may be expanded to larger surfaces if necessary before the 5 day endpoint.
24. Cell work-up and flow cytometry measurement.
  a. Harvest cells from the 48 well plate.
    i. Aspirate the medium from each well.
    ii. Wash the cells with 100 μL of PBS per well. Avoid rinsing the cell layer to avoid detachment.
    iii. Add 50 μL of Trypsin-EDTA to all wells.
    iv. Incubate at 37°C to allow cell detachment (5 minutes for HEK293T cells).
    v. Add 200 μL of complete medium, and gently resuspend cells.
    vi. Transfer the contents of each well to a BD Falcon U bottom plate.
    vii. Centrifuge the U-bottom plate(s) at 500 x *g* at 18°C–24°C for 5 minutes.
    viii. Carefully remove the supernatant using a multichannel pipette. Make sure not to disturb the pellets.
    ix. Add 200 μL of PBS to each well and resuspend by gently pipetting up and down 5x.
    x. Centrifuge the plates at 500 x *g* at 18°C–24°C for 5 minutes.
    xi. Carefully remove the supernatant using the multichannel pipette. Make sure not to disturb the pellets.
    xii. Add 200 μL of 1% paraformaldehyde to each well and resuspend by gently pipetting up and down 5x.
      ***Note:*** When fixing eGFP-positive cells with 1% PFA, it is recommended that flow cytometry analysis is performed on the same day. Longer storage of fixed cells, especially in higher PFA concentrations, is not recommended as the eGFP and BFP signals may diminish over time and cellular autofluorescence may increase.
    xiii. Incubate for 15–30 minutes at 4°C to fix the cells.
    xiv. Centrifuge the plates at 500 x *g* at 18°C–24°C for 5 minutes.
    xv. Carefully remove the supernatant using the multichannel pipette. Make sure not to disturb the pellets. Add 200 μL of PBS to each well and resuspend by gently pipetting up and down 5x.
    xvi. Centrifuge the plates at 500 x *g* at 18°C–24°C for 5 minutes.
    xvii. Carefully remove the supernatant using the multichannel pipette. Make sure not to disturb the pellets.
    xviii. Add 200 μL FACS buffer to each well and gently resuspend by pipetting up and down 5x. As a FACS buffer, we recommend a 1% BSA and 2 mM EDTA in PBS without Ca2+/Mg2+ buffer as described above.
  b. Measure cell fluorescence by flow cytometry.
    i. Prepare the flow cytometer according to the instrument’s specifications.
    ii. For eGFP, use a blue laser (e.g. 488 nm) and any filter set able to measure around its maximum emission of 510 nm.
    iii. For BFP, use a violet laser (e.g. 405 nm) and measure using a filter suitable able to measure around its maximum emission of 440 nm.
    iv. Additionally set up the run to acquire forward scatter (FSC) and side scatter (SSC) area and height.
    v. (Optional after first time set-up) First run untreated eGFP^+^ control cells to optimize the measurement settings for the forward scatter (FSC), side scatter (SSC) and fluorescent signals.
      ***Note:*** The FSC/SSC plot should have a large event cluster in the middle of the plot, while the eGFP signal should be in the top 25–30% of the detector limit to have good resolution for the eGFP signal. The BFP signal of these cells should be in the bottom 25–30% of the measuring range. It is important that as little events as possible exceed the lower and/or upper limit of the detection range. See also Figure 2 for an example of plots acquired using optimized measurement settings.
      **CRITICAL:** Compensation to correct for spectral overlap of BFP and eGFP may be required in some cases. This can be done by measuring eGFP^+^ cells and setting compensation in the BFP channel, when using a BD FACS Canto II flow cytometer, this compensation level is typically around 1–2%. We recommend to default to guidelines of the manufacturer of software used for flow cytometry analysis.

## Expected outcomes

**Figure 3:**
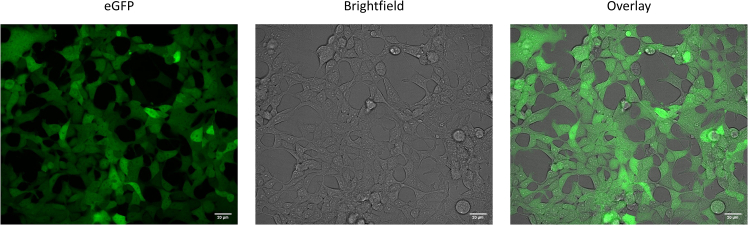
Fluorescence microscopy images of HEK293T-eGFP in cell culture The eGFP and bright-field channels are shown separately to show that cells express similar levels of eGFP, as shown in the overlay as well. Scale bar: 20 μm. Images were acquired using a Yokogawa CV7000 confocal microscope.

Fluorescent microscopy pictures are given in Figure 3 to show the eGFP fluorescence of HEK293T-eGFP cells growing in normal culturing conditions. This reveals a high signal as the exposure time of the cells to the laser was kept minimally low at 100 ms. Such microscopy confirms the successful transduction of the cells after 2–3 days.

We have utilized the eGFP to BFP model for a variety of research questions.^2^^,^^13^ Typical flow cytometry plots are given in Figure 2, as well as the gating strategy that we utilized in our work.

An example of processed data for a specific research question is given in Figure 1. We optimized HDR template length and concentration in ProDeliverIN CRISPR-Cas9 transfections for HEK293T-eGFP cells. The template sequences are noted in Table 1. Data was analyzed using Graphpad PRISM version 10.4.2 using a 2-way ANOVA and Tukey’s multiple comparisons test on the row means (RNP:template ratio) and column means (template length) when significant effects were found. Figure 1 shows that both template length and concentration significantly influence the relative HDR incidence (p < 0.0001 for the ANOVA, not shown in the graph). The post-hoc analysis shows that an increase from 86 to 166 nt does not have a significant effect on the relative HDR incidence. Analyzing the template concentrations in this dataset reveals that a 1:1 ratio of RNP to template yielded a significantly lower gene editing efficacy compared to the other ratios, and that there was no significant difference for the other ratios (shown in graph). Analysis of the other gene editing outcomes was not deemed needed in this study.

**Table 1:** HDR templates for mutation of eGFP to BFP with varying lengths of the homology arms

Length (nt)	Sequence
26	gaccaccctgaGcCacggcgtgcagt
46	ccaccctcgtgaccaccctgaGcCacggcgtgcagtgcttcagccg
66	gtgccctggcccaccctcgtgaccaccctgaGcCacggcgtgcagtgcttcagccgctaccccgac
86	caagctgcccgtgccctggcccaccctcgtgaccaccctgaGcCacggcgtgcagtgcttcagccgctaccccgaccacatgaagc
166	cctacggcaagctgaccctgaagttcatctgcaccaccggcaagctgcccgtgccctggcccaccctcgtgaccaccctga-GcCacggcgtgcagtgcttcagccgctaccccgaccacatgaagcagcacgacttcttcaagtccgccatgcccgaaggctacgt

The nucleotides containing the mutations are capitalized for clarity.

Thus, optimally, a template of 86 nt is used (6 nt for the mutation to BFP; 40 nt homology arms around these mismatched nucleotides) at a 2:1 ratio of template to SpCas9. This gives the highest relative HDR incidence for the least amount of DNA for these cells and transfection reagents. Relative HDR incidences around 10-25% of gene editing events are to be expected, and are in line with comparable studies on other genetic targets.^16^^,^^17^

## Quantification and statistical analysis

The data acquired from the flow cytometer provides insight into both gene knock-out and gene correction pathway activation. Using these values, we can calculate the total gene editing efficiency, as well as the relative incidence of HDR pathway activation.

First, the data needs to be gated to select cells and exclude (cellular) debris, followed by selection of single cells by excluding doublets. The plots in Figure 2 were generated using Flowlogic, however the general analysis strategy described below is applicable to other software as well. Import the raw .fcs files collected from the flow cytometer into the analysis software. Following this, group all controls and conditions with similar treatments. In the FSC(A) vs SSC(A) plot, gate the appropriate cell population, and exclude smaller cell debris fragments and larger cell aggregates if present (as shown in Figure 1A, I). Select single cells based on pulse geometry gating by plotting either FSC(A) vs FSC(H) or SSC(A) vs SSC(H) and draw a linear gate containing single cells (as shown in Figure 2, II).

After gating for single cells, plot the eGFP and BFP channels against each other, preferably using a dot plot or density plot graph. The fluorescent signal bleed-over should be negligible, as the BFP signal is relatively weak and barely emits in to the eGFP channel. All BFP-positive cells should be negative for eGFP. If this is not the case, either a longer recovery time after Cas9 delivery is required, or a cell line has been generated with multiple integrations in the cellular genome (see troubleshooting 1). In the eGFP+ controls without gene editing, draw a gate with low BFP signal (at the height of the highest cells in the plot and below) and low eGFP signal (bordering the large cluster at a high eGFP signal). Name this gate eGFP- / BFP-. In the same plots, draw a gate above the eGFP- / BFP- gate. Name this gate eGFP- BFP+. All plots now have gated populations for gene knock-out (eGFP- BFP-) and gene correction (eGFP- / BFP+). An example is given in Figure 2, III. It is recommended to confirm the gates for negative fluorescent signals by analyzing measurements of non-fluorescent (untransduced) control cells.

From this data, calculate the percentage of the eGFP- / BFP- gate and the eGFP- / BFP+ gate for all samples. Additionally calculate the percentage of eGFP- BFP- cells in the untreated eGFP+ controls and subtract this “blank” from all gene-edited conditions. This negative population, which is commonly present at a low percentage, can yield false positives for gene knockout if not corrected for. Repeat this blank subtraction for the eGFP- BFP+ gate, to subtract potential false positives within this gate for all samples as well.

After correction, these populations represent the “Absolute gene knock-out” (eGFP- / BFP-) and “Absolute gene correction” (eGFP- / BFP+) populations. To calculate “Total Gene Editing”, add up these percentages. Finally to calculate the “Relative gene correction” incidence, calculate the following:

Relativegenecorrection=Absolutegenecorrection/totalgeneediting∗100%

These values can be plotted and statistically compared for different gene editing conditions and provide insight into the specificity of HDR activation.

## Limitations

eGFP as model protein for gene editing has some limitations. First and foremost, the design in this work is all based on the use of SpCas9, which recognizes the NGG PAM sequence.^13^ Other Cas protein isotypes may have different PAM constraints which would require new designs for the guide RNA and template sequences. This can be done for example using the open access Benchling CRISPR tool (available from: https://www.benchling.com/crispr). It is recommended to test 3 to 5 guides to find one that has a high on-target activity. This model may be less suitable for Cas isotypes in which the DSB site is far away from the amino acids to be edited, as HDR efficiency goes down with higher distance.

The HDR template needs to be redesigned as well to include alternative silent PAM-inactivating mutations, required to increase HDR efficiency. In the case of PAM-independent nucleases, such as zinc fingers nucleases or TALENS, this additional mutation can be omitted.^18^^,^^19^ Beside the sequence we recommend similar HDR template length and concentrations as described in this study. If PAM-deactivating mutations are considered to be included in the HDR template, it is important to assess that these mutations do not induce any additional unwanted amino acid changes.

**Figure 4:**
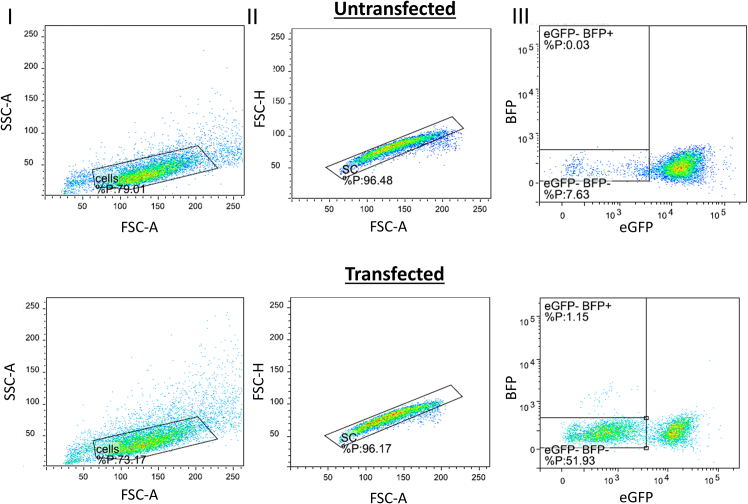
Typical gating strategy and eGFP genome editing results in Hepa 1-6-eGFP cells Gating for untransfected (top) or Cas9/sgRNA/HDR template transfected (bottom) Hepa 1-6 eGFP cells. Gating is shown for cells (I), single cells (II) and the analysis of the fluorescent populations (III).

**Figure 5:**
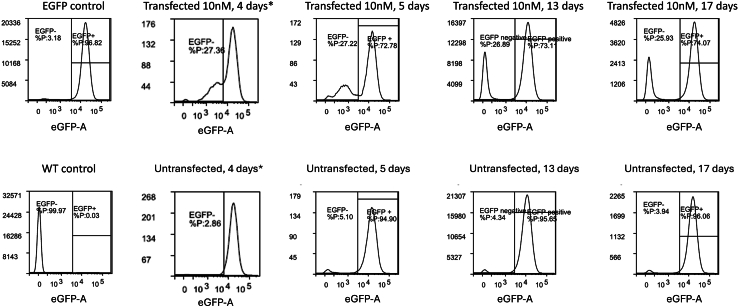
eGFP mutation stability shown as flow cytometry event histograms over time Cells were kept in culture for the noted amount of days. ∗: The data for 4 days of incubation were taken from a biologically independent transfection experiment than the other plots.

As the turnover of eGFP protein is slow, the knock-out efficiency is ideally measured after at least 5 days for HEK293T cells, as well as Hepa 1-6 and HepG2 in our experience. To demonstrate that this read-out is suitable for multiple cell lines, representative data of a Cas9 Hepa 1-6 eGFP cells is shown in Figure 4. The gating strategy described earlier for HEK293T eGFP cells (Figure 2) is applicable to this cell line as well, showing the broader applicability of this model for other stable cell lines. This creates technical challenges, as the cells need to be kept alive for a long timeframe. However, the mutation is stable, as it is made at the genomic level, and as such the effects on expression of eGFP and BFP are consistent between passages. This makes it possible to expand the treated cells to a suitable number for further experiments as shown in Figure 5. This timeline may be a concern if the cells do not tolerate passaging or require expensive culture media however.

Another drawback is that blue fluorescence is used as a positive readout in this model. Cells often exhibit autofluorescence in the lower wavelengths when they are in distress, which could yield false positives if this is not corrected for. This may be especially problematic when using (transfection) reagents that may induce cellular senescence or toxicity or when screening compounds which might induce blue autofluorescence.^2^ Proper controls are therefore recommended to compensate for such effects. For example, by using a non-targeting sgRNA which does not target eGFP, the effects of RNP transfection itself on fluorescence can be excluded. For further validation of the eGFP to BFP phenotypical data we recommend sequencing based readouts such as TIDER.^20^

Additionally, the intensity of the fluorescent signal of BFP is relatively weak, as compared to other fluorescent proteins such as eGFP. As such we strongly recommend using lentiviral transfer plasmids that contain a promotor for the eGFP gene with strong expression, such as EF1a or CMV promotors. A potential solution to address this is to use an inverted readout compared to this work, where the BFP mutant described here can be mutated to eGFP. The methodology presented in this chapter would be applicable to that model as well, except that a different lentiviral transfer plasmid and slightly redesigned sgRNA and ssODN template are necessary. Other blue fluorescent proteins than BFP are described as well, but as they would require more mutations at multiple locations in the protein, these proteins are less suitable for studying point mutations.

Finally, translation of the findings from constitutively active genes such as here eGFP to other genomic targets requires verification. An advantage of the protocol presented here is the high on-target genome editing seen on the eGFP gene, as well as a sensitive and convenient read-out. Applying these findings to endogenous genes will require guide RNA and template DNA optimization for the specific target locus, and in our experience this may lead to differences in gene editing efficiency as compared to the described fluorescent reporters. This model therefore presents a valid, robust and scalable method to study Cas9-gene editing, but directly translating these findings to specific endogenous genomic targets may require additional optimization and validation.

## Troubleshooting

### Problem 1

Double positive (eGFP+/BFP+) cells, or heterogeneous eGFP expression levels (related to steps 7-9 of Lentiviral Transduction and step 2 of Cell work-up and flow cytometry). This can be caused by multiple integration events of the eGFP gene or phenotypic differences in the eGFP expression between clones, respectively. The former is a larger problem, as it may lead to double positive eGFP+/BFP+ cells after gene editing due to part of the eGFP genes in the cell being edited.

This complicates analysis later in the protocol resulting in potential underestimation of NHEJ-mediated eGFP knockout. Moreover it can cause double eGFP and BFP positive cells to show up in analysis, interfering with NHEJ:HDR ratio calculations.

### Potential solution

• The difference between multiple integrations or phenotypic differences between cells can be determined by quantifying the amount of integrated vectors, for example by RT-qPCR.^21^ In case of multiple integrations, consider repeating the transduction at a lower MOI as noted as NOTE under Lentiviral transduction. the MOI of the transduction can be optimized to 0.01–0.1 to prevent multiple integration events as much as possible.
• Observation of eGFP+/BFP+ cells can be the result of incomplete degradation of the eGFP protein after genetic editing due to the high stability and long half-life of eGFP,. In this case, a longer incubation time after transfection is recommended to allow its degradation. An assay to follow the gene-edited cells over time, such as the data in Figure 5, is suggested in that case.
• An additional monoclonal selection method can be used to normalize the eGFP levels in the case of phenotypic differences. This can be achieved through fluorescent-activated cell sorting (FACS) according to protocols outlined in this work,^22^ or by limited dilution (for example outlined here: https://www.addgene.org/protocols/limiting-dilution/). In this way cells are seeded as single cells per well to grow out to colonies to achieve monoclonal selection. Using conditioned medium, supernatant from the previous culture, may aid in stimulating proliferation. Isolating 3 clones and pooling them is recommended to avoid issues due to clonal drift.

### Problem 2

No fluorescent cells are seen after transduction (Related to step 7 of Lentiviral Transduction).

After cell transduction, the eGFP fluorescence should be visible after 48–72 hours of incubation. If this is not the case, the transduction may have failed, which can be due to a low infectivity or titer of the lentivirus.

### Potential solution

It is recommended to determine the multiplicity of infection (MOI) on the cell type that is intended for transduction. There are several potential solutions, in order of ease.

• It may be necessary to add a higher volume of lentiviral supernatant to not dilute it using fresh medium.
• The infectivity of the lentiviral supernatant can be increased by pre-incubation using polybrene, see also the following Denning et al.^23^
• It may help to concentrate the supernatant by ultracentrifugation, see also the following Brown et al.^24^

### Problem 3

Cells are lost after antibiotic selection of the lentivirally transduced cells. Even if the cells show eGFP expression, they can be lost due to unoptimized antibiotic selection. In this work we provide example incubation times and concentrations for HEK293T cells and puromycin selection, but other cell lines or eGFP expression constructs may require optimization.

### Potential solution

An antibiotic kill-curve can provide an accurate concentration for the antibiotic selection. A concentration in which non-transduced cells start to show cytotoxicity is suitable for selection.^25^

The required culture time with the selection antibiotic is strongly dependent on the selection antibiotic. It is recommended to default to the manufacturer’s guidelines for the selection antibiotic. In the case of puromycin selection, we generally adhere to at least 5 days of selection. Throughout the antibiotic selection, cells can be cultured as normal. However, we recommend that the cell culture medium is refreshed every 2–3 days supplemented with appropriate selection antibiotics, e.g. 2 μg/mL puromycin. Afterward, it is not uncommon to culture the cells in a decreased selection antibiotic “maintenance” concentration, which is often a 2-fold decrease of the lowest selection concentration. Again, as this may differ among selection antibiotics, we recommend to default to the manufacturer’s guidelines where appropriate.

### Problem 4

Low cell numbers in flow cytometry (<10,000 total events in the well), related to steps 1 and 2 of the cell workup and flow cytometry section.

After transfection of the CRISPR-Cas9 reagents and potentially washing steps, it is common to lose cells. Losing cells in these early stages will lead to lower confluency during the maintenance phase in Steps, which in turn leads to a very low cell number during flow cytometry which may lead to failure of the experiment.

### Potential solution

Be very gentle when handling the transfected cells. Especially washing steps can lead to loss of the cell monolayer in the 96-well plate format. Assuming that the edited cells have the same chance to be lost as not-edited cells, it is possible to simply grow the cells out to higher numbers prior to trypsinization and seeding in a fresh plate. Alternatively, if the flow cytometer is housed in a biosafety laboratory space, the cells can be analyzed without the use of a fixation step, limiting the amount of washing steps resulting in decreased cell loss.

### Problem 5

Very low genome editing efficiency (BFP+ AND eGFP-) (Related to steps 1-4 of transfecting CRISPR-Cas9 reagents in cells and step 2 of cell work-up and flow cytometry).

It is possible that transfection using the concentrations and reagents described in step 1 of the transfection section, which were optimized for HEK293T cells and shown to work in Hepa 1–6 cells as well,^1^^,^^2^^,^^26^ are unsuccessful.

### Potential solution

Transfection conditions need to be optimized for new cell lines and culture conditions used. Deviating from the manufacturers protocols can be necessary, for example by providing cells with a higher Cas9 RNP concentration. Other transfection reagents or methods, such as nucleofection, can be more successful depending on cell line and experience.

### Problem 6

Very low HDR efficiency (BFP+) while gene knock-out efficiency is high (high eGFP- BFP-) (related to step 2 of cell workup and flow cytometry).

The incidence of HDR can be low, especially in complex cell models and non-proliferative cells. This can lead to a low amount (<1%) of BFP+ cells in the analysis.

### Potential solution

Acquire more than 10,000 events to ensure that BFP+ population percentage is reliable. Potentially it is necessary to grow the cells to a confluent well in a 24 or 12 well plate to ensure enough cells are present for analysis. Further optimization of HDR template DNA may be needed as well if a low BFP+ population is noted.

## Resource availability

### Lead contact

Further information and requests for resources and reagents should be directed to and will be fulfilled by the lead contact, Olivier Gerrit de Jong (o.g.dejong@uu.nl).

### Technical contact

Technical questions on executing this protocol should be directed to and will be answered by the technical contact, Danny Wilbie (dannywilbie@gmail.com).

### Materials availability

This study did not generate new unique reagents.

### Data and code availability

This study did not generate/analyze datasets or code.

## Acknowledgments

The graphical abstract was prepared using Biorender.com.

This research was funded by the 10.13039/501100003246Netherlands Organisation for Scientific Research (NWO) Talent program VICI, grant number 865.17.005.

## Author contributions

D.W. set up the main protocol and performed the experimental work and preparation of the initial manuscript, as well as revisions to the manuscript. E.M. supervised the project and provided feedback and revisions on the manuscript. O.G.d.J. supervised the project, helped in writing of the manuscript, provided protocols, and prepared materials used in this work.

## Declaration of interests

O.G.d.J. is on the advisory board of The Organoid Company, Surry Hills, Australia.
